# Novel Molecular Vehicle-Based Approach for Cardiac Cell Transplantation Leads to Rapid Electromechanical Graft–Host Coupling

**DOI:** 10.3390/ijms241210406

**Published:** 2023-06-20

**Authors:** Aleria Aitova, Serafima Scherbina, Andrey Berezhnoy, Mikhail Slotvitsky, Valeriya Tsvelaya, Tatyana Sergeeva, Elena Turchaninova, Elizaveta Rybkina, Sergey Bakumenko, Ilya Sidorov, Mikhail A. Popov, Vladislav Dontsov, Evgeniy G. Agafonov, Anton E. Efimov, Igor Agapov, Dmitriy Zybin, Dmitriy Shumakov, Konstantin Agladze

**Affiliations:** 1 Laboratory of Experimental and Cellular Medicine, Moscow Institute of Physics and Technology, Institutskiy Lane 9, 141700 Dolgoprudny, Russia; 2 M.F. Vladimirsky Moscow Regional Clinical Research Institute, Schepkina St. 61/2, 129110 Moscow, Russia; 3 Almetyevsk State Oil Institute, 2 Lenina St., 423450 Almetyevsk, Tatarstan, Russia; 4 Nanobiomedicine Division, Sirius University of Science and Technology, 1 Olympic Ave, 354340 Sochi, Russia; 5 Academician V.I. Shumakov National Medical Research Center of Transplantology and Artificial Organs, Ministry of Health of the Russian Federation, 1 Schukinskaya St., 123182 Moscow, Russia

**Keywords:** cardiovascular, cell culturing, electrophysiological coupling, electrospinning, engraftment, microcarriers, optical mapping, transplantation

## Abstract

Myocardial remodeling is an inevitable risk factor for cardiac arrhythmias and can potentially be corrected with cell therapy. Although the generation of cardiac cells ex vivo is possible, specific approaches to cell replacement therapy remain unclear. On the one hand, adhesive myocyte cells must be viable and conjugated with the electromechanical syncytium of the recipient tissue, which is unattainable without an external scaffold substrate. On the other hand, the outer scaffold may hinder cell delivery, for example, making intramyocardial injection difficult. To resolve this contradiction, we developed molecular vehicles that combine a wrapped (rather than outer) polymer scaffold that is enveloped by the cell and provides excitability restoration (lost when cells were harvested) before engraftment. It also provides a coating with human fibronectin, which initiates the process of graft adhesion into the recipient tissue and can carry fluorescent markers for the external control of the non-invasive cell position. In this work, we used a type of scaffold that allowed us to use the advantages of a scaffold-free cell suspension for cell delivery. Fragmented nanofibers (0.85 µm ± 0.18 µm in diameter) with fluorescent labels were used, with solitary cells seeded on them. Cell implantation experiments were performed in vivo. The proposed molecular vehicles made it possible to establish rapid (30 min) electromechanical contact between excitable grafts and the recipient heart. Excitable grafts were visualized with optical mapping on a rat heart with Langendorff perfusion at a 0.72 ± 0.32 Hz heart rate. Thus, the pre-restored grafts’ excitability (with the help of a wrapped polymer scaffold) allowed rapid electromechanical coupling with the recipient tissue. This information could provide a basis for the reduction of engraftment arrhythmias in the first days after cell therapy.

## 1. Introduction

The constant accumulation of local myocardial damage cannot be compensated for by the regenerative potential of the heart tissue [1], which leads to high mortality from cardiovascular diseases. Transplants made from cultured cardiac cells are believed to play an important role in regenerative medicine of the heart [2,3,4], particularly in repairing the cardiac conduction system [5,6]. The easiest and simplest way to introduce healthy cells into the damaged area is to inject solitary cells in the form of cell suspension [7]. Unfortunately, after numerous studies, this method appeared to be much less efficient than expected [4,8]. The injection of stem cells does not guarantee that they will differentiate into right cardiomyocytes, and the injection of cultured cardiac cells does not guarantee their survival and functional merging with the host tissue. In the latter case, the main problem is that solitary cardiomyocytes lose their structure and become very fragile and unexcitable. As known from cell culture [9], the cell adhesion and recovery of cardiomyocytes’ structure and excitability take several hours, during which the cells should rest and not be subjected to any mechanical stress. The latter condition is difficult to fulfill in living cardiac tissue. The way to overcome fragility problems is to deal with cells on a scaffold, allowing them to maintain their structure. However, common cultured cell patches [10] are hardly appropriate for such a simple method of delivery as injection and require surgical intervention.

Thus, there are two common ways of delivering cells to the host heart tissue: the injection of dispersed solitary cells and the integration of cells as part of tissue-engineered constructs. The most significant difference between these two transplantation methods is host–graft electrophysiological coupling [11]. Gerbin et al. demonstrated host–graft electrophysiological coupling with dispersed cells, but not tissue-engineered constructs, confirmed with optical mapping experiments [12]. Dispersed cells can directly come into contact with excitable host tissues [12]. Therefore, dispersed cells easily become a substrate of arrhythmia due to the prolonged and stochastic formation of electromechanical coupling. For example, the injection of suspended iPSC-CMs into allogenic heart tissue (non-human primate, histocompatible primate-induced pluripotent stem cells) results in acts of prolonged ventricular tachycardia [8,13,14]. In contrast, the engraftment of iPSC-CM patches did not lead to the formation of stable electrophysiological coupling between the rat heart and the graft [12,15,16]. The peak presence of arrhythmias after cell injection is usually observed in the first two days after transplantation [8,10,13], which is close to the above estimate of the time required for the formation of gap junction (GJ) coupling [9]. Furthermore, dispersed cells can leave the injection site and have a low survival rate [7], whereas a significant proportion of the cells do not graft into the host tissue [17]. The latter is due to the fact that the continuous presence of adhesion sites is necessary for the normal functioning of iPSC-CM, and in its absence, the viability and functionality of the cardiomyocyte are limited [18]. On the contrary, tissue-engineered constructs demonstrate improved survival and cell viability after transplantation, but the shape and size of the graft impose restrictions on the method of delivery to the host tissue and challenge electrophysiological coupling [15,16]. In addition to the approaches mentioned, there is an intermediate option in the form of using hydrogels to hold the delivered cells. However, similarly, the cells surrounded by the gel cannot fully integrate into the electromechanical syncytium of the host heart tissue and between each other [19,20]. As a result, there is controversy as to when the presence of a scaffold is required for cell survival but makes cell delivery invasive and prevents electromechanical coupling. In addition, the complete absence of a scaffold or any substrate makes electro-mechanical coupling chaotic because of the restoration of cell excitability and prolonged formation of GJ, accompanied by engraftment arrhythmias.

We propose a method employing the advantages of a polymer nanofiber scaffold with the feasibility of cell delivery. For this purpose, the supporting cell “scaffold” represents a number of isolated fragments of polymer nanofibers capable of carrying solitary cells. As a material for polymeric nanofibers, we used poly-L-lactide (PLLA), a biocompatible and biodegradable polymer widely used for medical purposes: the resulting fibers turned out to be thinner than native fibroin fibers [21], and human fibronectin (HFN) was used to optimize the adhesive properties of the fiber surface [18]. It should be noted that making the optimal choice of polymer composition is a complex and global task [22]. An approach to a polymer nanofiber as a sufficient cell substrate has been previously demonstrated in vitro. Polymer fiber fragments of subcellular sizes coated with HFN are a satisfactory substrate for the cultivation of both neonatal rat ventricular cardiomyocytes (NRVMs) and induced pluripotent stem cell-derived cardiomyocytes (iPSC-CMs) [18,21], with the latter having the greatest potential for clinical use [4]. Cell adhesion to a single fiber leads to the appearance of a remarkable “sheath” structure, enveloping fiber, thus substantially increasing the contact zone and eventually forcing the cell to attach to itself [21]. Thus, the scaffold moves “inside” the cell (topologically remaining outside of the cell membrane, which is curved in such a way as to create an invagination to envelop the fiber with the cell). The main consequence of this cell–fiber interaction is the restoration of excitability and mechanical contractions [21] in a solitary cell that were lost during enzymatic methods of cell isolation [9,18]. As a result, the cell injection solution, instead of a suspension of solitary cardiomyocytes, represents a suspension of cells on compact carriers. Here, we directly tested the ability of novel nanofiber polymer scaffolds to mechanically bind transplanted cardiomyocytes (NRVMs) and the host tissue, as well as to become a sufficient substrate for cell adhesion, thereby replacing cell culture isolation with the isolation of fragmented polymer fibers. This allows cells to be delivered without enzymatic disaggregation. The latter allows cells to restore excitability and contractility until the moment of transplantation, which initiates and accelerates the process of electrophysiological merging with the recipient tissue.

The current study aimed to address two principal gaps in knowledge. First, the proposed molecular machine design is an attempt to resolve the mentioned tensions between scaffold-based cell delivery approaches and noninvasive single-cell injection without scaffold. The interaction of cells and proposed scaffolds differs from previous approaches in that the necessary polymer mass (nanofiber) is wrapped by the cell. When the diameter of such a mobile scaffold is minimized, the graft also acquires the main strength inherent in conventional solitary cells—direct contact with the host tissue. The combination of the simultaneous presence of the scaffold while eliminating its usual shortcomings is the achievement of the proposed approach. The fundamental feasibility of such a delivery method opens up a new way to circumvent the traditional problems of cardiac tissue cell therapy. The second question to be addressed is: What will be the scenario of electromechanical coupling? The presence of a mobile microscaffold makes the cell excitable by the time of first contact with the tissue, in contrast to the solitary cell [18]. Recent studies have actualized the ephatic mechanism of electromechanical coupling (without GJ formation), both during excitation conduction through the tissue [23] and in the context of host–graft interactions [13]. An ephatic coupling (EPC) becomes possible at the moment when both elements are excitable [11]. Previously, we suggested that the wrapped fiber microcarrier could become a catalyst for the rapid emergence of EPC prior to the formation of GJs [18]. In this work, we set out to observe directly whether the occurrence of EPC in vivo is possible. For this purpose, we created the conditions for observing electromechanical synchronization immediately after the moment of cell delivery (rather than at a daily interval, as in other studies involving optical mapping [8,10]). Achieving host–graft electromechanical synchronization before GJ formation may be the key to eliminating acute engraftment arrhythmias.

## 2. Results

### 2.1. Polymer Carriers for the Isolation and Transplantation of Single Cardiomyocytes

**Figure 1:**
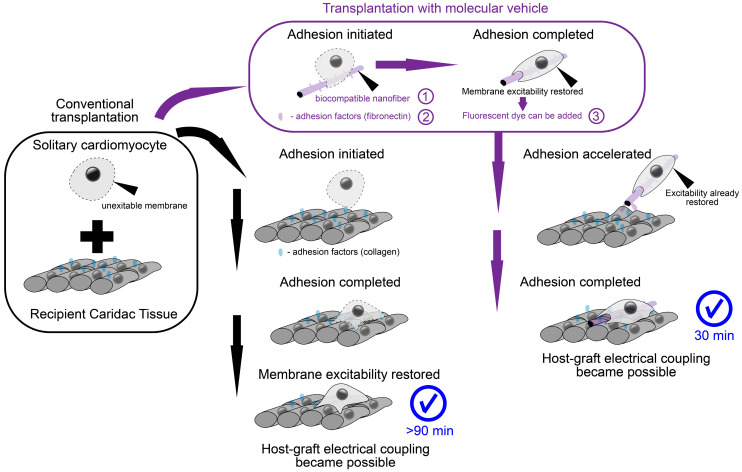
Schematic components of the molecular vehicle (microcarrier) and experiment description. Black arrows show the usual way of cell transplantation or injection into cardiac tissue. Purple arrows show a new method of cell transplantation that accelerates the functional integration of the graft into the host tissue. The three key components to speed up the integration process are marked with purple numbered circles.

In the present work, we studied host–graft interaction during cardiomyocyte transplantation into rat hearts using nanofiber microcarriers. The scheme in Figure 1 shows the components included in the nanofiber microcarrier designed for the cultivation of excitable isolated cardiomyocytes. The first and main component is a fragment of polymer fiber with a thickness of approximately 0.85 µm ± 0.18 µm (*n* = 20), obtained by electrospinning the PLLA solution. The second element is the protein coating of the polymer fiber by placing the fibers in an HFN solution for 24 h. This resulted in protein deposition on the fiber surface, which manifested itself in the ability of cardiomyocytes to adhere to fibers of a given thickness. The third component was a fluorescent label used for optical tracking. Samples that had all three components and adhered cells were used in the experiment described in this paragraph and were designated as microcarrier, protein, and cell (MPC)-samples. Microcarriers without cells are referred to as MP-samples, and single cells without microcarriers are referred to as C-samples. The dye used for visualization is specified in the text in each individual case.

**Figure 2:**
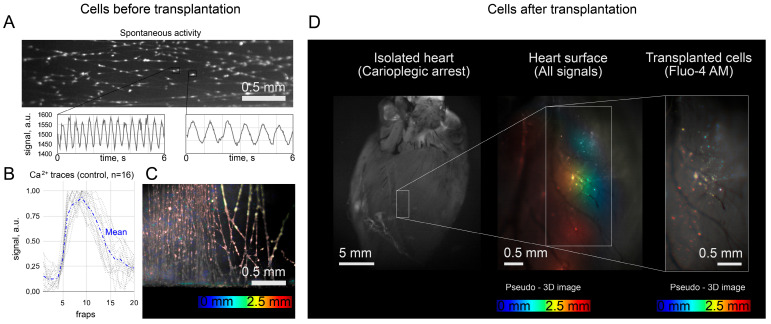
(**A**) The spontaneous activity of such NRVM cells on polymer microcarriers before transplantation. (**B**) Spontaneous excitation signal patterns (Fluo4-AM) for 16 cells (4 different samples) with spontaneous excitation frequencies in the range from 0.5 to 1.5 Hz. The signal was averaged within 3 pixel × 3 pixel frame at 34 frames per second. (**C**) Pseudo-3D image of seeded NRVMs begore transplantation. (**D**) The site of fiber application on the surface of the heart and its pseudo-3D image (color-coded projection) with transplanted cells. The color gradient (from red to blue) indicates the relative location of the surface points along the *Oz* axis, perpendicular to the figure plane.

First, we tested the interaction of cells cultured on fragmented nanofibers (Section 4) with the surface of the isolated heart. A freshly prepared suspension of solitary NRVMs was seeded on a grid of nanofibers attached to blocks of Poly-dimethylsiloxane (PDMS). The cultivation of cells for 24 h led to the following: single NRVMs adhered to the surface of nanofibers and restored spontaneous activity, which was recorded using Fluo4-AM dye 24 h after seeding. The mechanism of cell adhesion to thin nanofibers with restoration of excitability was studied earlier [21] and illustrated in Figure 1 as the basis of the developed approach to cell delivery. Convincing evidence of cell adhesion is the restoration of spontaneous cell excitability, the spontaneous excitation signal patterns are shown in Figure 2A,B. The average signal (mean) was obtained on the basis of 16 calcium traces obtained from 16 different cells (4 different samples) with spontaneous excitation frequencies in the range from 0.5 to 1.5 Hz. The signal was averaged within 3 pixel × 3 pixel frame at 34 frames per second. An important condition was the absence of coupling between NRVMs (Figure 2A): the cell density was chosen in such a way (Section 4.1) that the cells did not form a conductive tissue. Otherwise, the establishment of coupling between the cell and the surface of the heart as the only source of external excitation (Figure S1, Supplementary Video S1) would be impossible (see Section 3).

Thus, by the time of optical mapping, it is assumed that each cell has carried out the process of adhesion (initiated by HFN) with enveloping the polymer surface and, thereby, has occupied a local fragment of the fiber. Figure 2C shows a pseudo-3D image of cells on fibers before transplantation: the bottom of the cup is displayed here in blue (faintly visible in the gaps between the fibers), and the plane of the nanofibers grid with attached cells in shades of red and yellow. In this form, the cells are located on the fibers (Figure 2A,C) and are equipped with a fluorescent marker (Figure 2A,B), Fluo4-AM, being ready for transplantation.

An isolated rat heart was used as the recipient tissue (Figure 2D). The preparation for perfusion consisted of the following steps detailed in the methods: anesthesia of the animal, extraction of the heart with Langendorff cannulation, flushing with Tyrode’s solution, and cardioplegic arrest with Normacor and oxygenated solution of Tyrode in 1:4 proportion. Under such conditions, without changing the temperature regime, a temporary (30 min) cardiac arrest was achieved to simplify the adhesion of the graft to the immobilized surface. Immediately after cardiac arrest, cells were transferred and the fiber ends were separated from the PDMS blocks (Section 4.1), then from that moment the cells remained attached to the fiber fragments (free of PDMS blocks), and the fiber and its enveloping cell were in direct contact with the surface of the heart.

From that moment on, the heart and cells were kept in a liquid medium at physiological temperature (Figure 2D). Although the processes of cell excitation were stopped by the cardioplegic solution (according to the hyperkalemic scenario), there were no obstacles to cell spreading and adhesion. As a result, attachment of the graft to the surface of the heart was observed: the location of cells on the surface was fixed and remained unchanged despite the movement of the perfusate and, looking ahead, remained after the restoration of the contractile activity of the heart. The right panel in Figure 2D shows the arrangement of cells (each color spot represents a stationary Fluo4-AM signal from a single cell) on the surface of a recipient heart. In contrast to the similar pseudo-3D image from Figure 2C, the cells are located not in the same plane, but in accordance with the curvature of the surface of the heart.

The perfusate was replaced with oxygenated Tyrode’s solution (37 °C) 30 min after the NRVMs transplantation. During the next 10 min, the recovery of spontaneous cardiac activity was recorded. First of all, the restoration of spontaneous activity of NRVMs occurred (Figure S1B). Recovery of cardiac contractions after transplantation of NRVMs and washing of cardioplegia was successful in 3 out of 4 repetitions performed (Heart #2–4), while spontaneous cell activity was restored in all cases.

**Figure 3:**
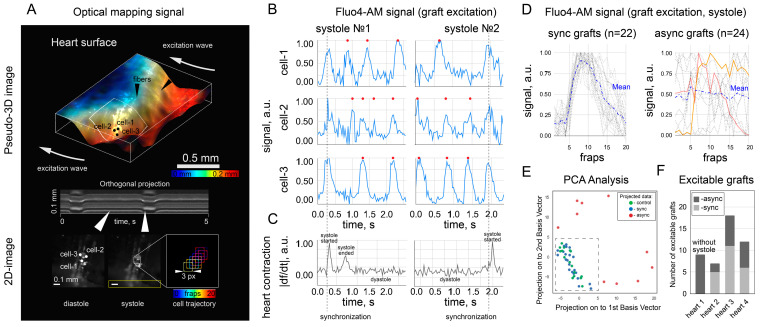
Optical mapping of the transplanted cells on the surface of the heart and synchronization of the cells with each other due to the excitation wave passing through the heart. Subfigures (**A**–**C**) illustrate in detail an example of the analysis procedure (on 3 cells out of 46 examined). (**A**) Color image of the pseudo-3D surface of the heart showing three excitable cells with spontaneous activity and unrelated to each other. The color gradient (from red to blue) indicates the relative location of the surface points along the Oz (vertical) axis. Systole and diastole are shown in the orthogonal projection of the area highlighted by the yellow rectangle. The white arrows link the video frame to the corresponding point in time on the orthogonal projection (systole and diastole); the lower right panel illustrates the trajectory of the displacement of the pixel frame during systole; the color shows the moment in time (frame) at which the frame occupied the selected place. (**B**) Fluorescence intensity (Fluo4-AM) in cells (cells 1–3) measured with a frame moving with the cell during systole. Red dots mark spontaneous grafts’ excitation during heart diastole. (**C**) The time derivative of the signal plotted from the orthogonal projection in (**A**). The growth of the derivative indicates the beginning of systole, and the stationary values indicate the onset of diastole. (**D**) Excitation (Fluo4-AM) signal patterns for 22 “sync” cells during heart’s systole is shown on the left. The signal was averaged within 3 pixel × 3 pixel frame at 34 frames per second. Signal patterns for 24 “async” cells during heart’s systole is shown on the right. Mean value is highlighted with blue dotted line. Two examples of “async” cells is highlighted with red and orange colors to demonstrate an incidental resemblance to the shape of the calcium trace. (**E**) Principal component analysis for control, sync and async signals. (**F**) Distribution of cells into sync and async groups in 4 conducted experiments.

The key subject of the study here was the calcium dynamics of NRVMs during cardiac systole. Electrical excitation of cardiac tissue in this case was considered as an external stimulus for NRVM cells attached to the heart during cardioplegic arrest. A necessary condition for recording the Fluo4-AM signal during the heart’s systole was the displacement of the pixel frame (by analogy with Figure 2B) following cell movement. The process of moving the frame is described in Section 4.4 and illustrated in Figure 3A (in particular, the lower right panel).

Figure 3A–C contain an illustrative example of Fluo4-AM signaling from several cells located on the surface of a beating heart (Heart #2). The signal of each cell is shown on Figure 3B and has a common time axis with Figure 3C, showing the beginning and end of heart systole. For each cell, there are several calcium traces during diastole (i.e., in a fixed reading frame, in complete analogy with Figure 2B), and these traces are marked with red circles at the top. Additionally, for each cell, a calcium trace is given, obtained by shifting the frame after the cell during systolic contraction of the heart; such traces are marked with a gray dotted line and are of key importance here. Thus, Figure 3B shows Fluo4-AM fluorescence intensity in the three selected cells, averaged within a 3 pixel × 3 pixel frame (resulted signal-to-noise ratio was 32 dB) moving with the cells during the systole phase. Altogether, graphs (Figure 3B,C) show that in systole, all three cells were synchronized with each other (and, consequently, with the excitation wave in the heart), although the rhythms of the spontaneous activity (e.g., during diastole) of these cells were initially different. That is, three single cells in systole were synchronized with the recipient heart. Hence, the recipient heart’s excitation became an external source of stimulation for all three transplanted cells. This example is used for illustration, since the effect is illustrated on several cells at once.

All registered (in 4 independent experimental replicates) calcium traces of cells during systole can be divided into two groups: “async” (asynchronous with respect to excitation of the heart) and “sync” (synchronous), where “async” represents NRVMs with only spontaneous activity (no synchronous excitation with the recipient heart), and “sync” represents NRVMs whose activity was synchronized with the excitation of the heart (Supplementary Videos S2 and S3). By definition, both cell types showed spontaneous calcium activity during diastole, but had different activity during systole. A cluster belonged to the sync group if its signal during systole had the same shape as the control signal obtained from cells before transplantation (Figure 2B). Figure 3D on the left shows such signals from 22 cells identified as sync: the average shape of the calcium trace is similar to the control signal from Figure 2B. The case with async clusters was more complicated: it is obvious that the frame shift following the cell during systole can register not only the occurrence of a calcium trace, but also random changes in the signal. A set of such random signals is shown on the right side of Figure 3D for 24 cells. The averaging of all signals among themselves was expected to be noise, however, individual cases could have a deceptive resemblance to control calcium traces (two examples are illustrated on Figure 3D).

To strictly formalize the division of clusters into async and sync groups, the following approach was implemented: for each studied cell during systole, a signal with a duration of 20 frames was recorded and represented as a 20-dimensional vector (each dimension is the average signal brightness in the frame on a given frame). The signals presented in this form were subjected to principal component analysis with a reduction in dimension from 20 to 2, the result of clustering is shown in Figure 3E. Signals from Figure 2B were also taken into consideration as control signals. The result of PCA analysis was the clustering of control signals with sync signals, which proves the presence of calcium excitation of sync cells during systole. At the same time, async signals were outside the cluster, therefore, they were an artifact from frame shift during systole. The summary statistics for the detection of sync and async cells in the 4 experiments performed are presented in Figure 3F and in Table 1.

### 2.2. Injection of Cells with Microcarriers into the Rat Heart In Vivo

**Figure 4:**
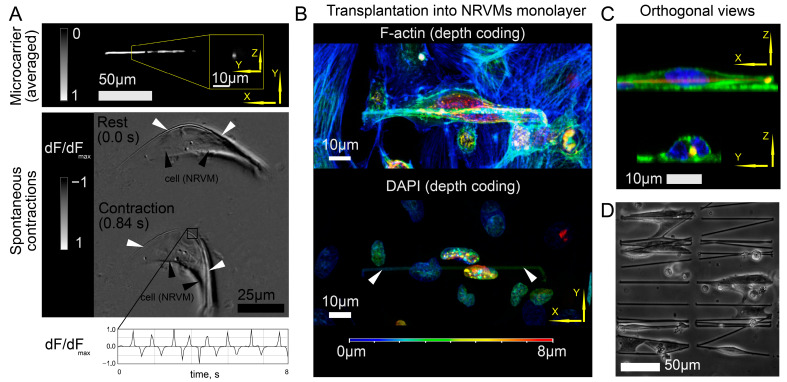
In vitro study of isolated cell functionality on short fragments of nanofibers intended for transplantation through an insulin needle. The white arrows point to polymer fibers. (**A**) The optical image of a microcarrier with a fiber segment length of approximately 90 μm and contractile cell on it. The lower half of the image shows the contractions of a single cell with absorbed fiber, and the upper half shows the characteristic dimensions of the fiber. The graph with white background shows a change in the dF/dF_max_ signal over time in an area selected by a white square frame. (**B**) Cell adhesion to a monolayer of cardiomyocytes with color-coded projection. The color scale indicates the coordinates along the *Oz* axis. (**C**) Orthogonal projections of the image shown in Figure 4B. (**D**) Seeding density of cells on microcarriers to obtain MPC-samples.

This section presents the results of the noninvasive external monitoring of cell engraftment delivered by injection using polymeric microcarriers. To simplify cell transport, we reduced the length of the microcarriers to 86.2 µm ± 5.2 µm (*n* = 50, the accuracy was limited by non-ideal fiber alignment during electrospinning), which allowed unhindered transfer of the suspension using an insulin syringe needle (both techniques of microcarrier fabrication are described in Section 4). The use of shorter fiber fragments did not affect the ability of the cells to recover their excitability and contractility. Figure 4A shows an averaged optical image of a microcarrier with a fiber fragment length of approximately 90 μm. Culturing a single NRVM cell on a microcarrier of this size showed that the cell also exhibited periodic excitability and mechanical contractions (Figure 4A).

Before injecting short microcarriers with cells (MPC-samples) into the rat myocardium, the samples were tested in tissue culture (in vitro). Figure 4B,C show cell adhesion to a monolayer of cardiomyocytes with color-coded projection (Figure 4B) and in orthogonal views (Figure 4C). These images show cell adhesion to the NRVM monolayer, with cell expansion toward the bottom. Thus, the cells retained the ability to adhere to and contract on the fibers, and the cell cytoskeleton retained plasticity without the loss of adhesion to the microcarrier. Figure 4D shows the cells plated on microcarriers before being transferred to an injection syringe. This suggests that not all of the fibers in the MPC-samples were covered with cells: estimated coverage efficiency was 45% ± 4% (*n* = 4).

**Figure 5:**
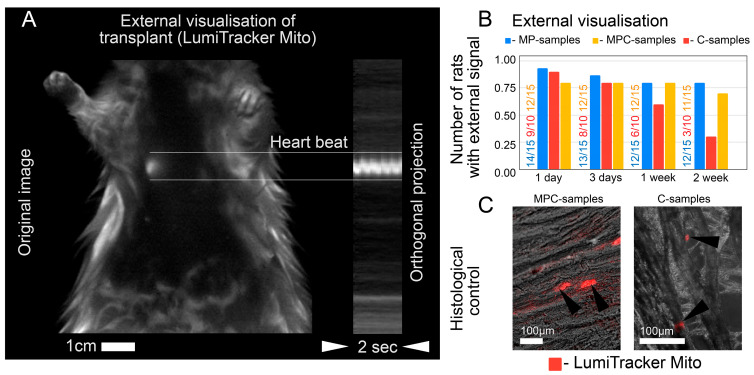
Observation of the results of the injection in vivo. The dye used in conjunction with polymer fibers allows fluorescence to be detected without dissection of the animal. (**A**) shows the visualization of the dye (cells + fibers) in a live rat with a beating heart. (**B**) showshow fluorescence changes over time: C-samples (red), MP-samples (blue), and MPC-samples (yellow). (**C**) Histological control shows an example of a heart section (60 µm) after MPC-sample (left) and C-sample (right) injections 14 days after operation. Black triangular arrows indicate grafts. Cells on the microcarriers were labeled with red fluorescent dye (LumiTacker Mito) before the injections.

All samples were tested in vivo using short operations on rats, as elaborated in the Section 4. By injection, we introduced the following samples, respectively: 10 rats for C-samples (cells), 15 rats for MP-samples (microcarrier with HFN protein), and 15 rats for MPC-samples (microcarriers, HFN, and adhered cells). Here, the C-samples and MPC-samples were supplied with LumiTracker Mito dye, whereas the MP-samples were supplied with BPD dye (due to the absence of cells in this group). To practice staining and test the in vivo mapping setup, we also injected each type of specimen into the paws of one to two animals per group. All such controls were visible after 24 h for all samples. In vivo imaging showed the heart beating, with the labeled samples in it for all rat groups (Figure 5A, technically mediated signal-to-noise ratio in raw video recordings was 13–19 dB). The fluorescent signal was subjected to a qualitative analysis to determine the persistence of the dye in the heart after injection (the possibility of quantitative analysis is discussed in the Section 3). A day after the operation, fluorescence in the heart was observed for the C-samples in 90% of the cases (9 out of 10 rats), for MP-samples in 93% (14 out of 15 rats), and for MPC-samples in 80% (12 out of 15 rats), as shown in Figure 5B. On the 3rd day after the injection, there was a sharp decline in the incidence of fluorescence for the C-samples (80%), which lasted up to two weeks. After two weeks, fluorescence was observed in only 30% of the rats from the group. Histological analysis confirmed the presence of grafts in the MPC-samples, with observed external fluorescence (Figure 5C). For 3 days after injections, the fluorescence of the MP-samples was registered in 86% of the rat group and that of the MPC-samples was similar (80%). Two weeks after the operation, fluorescence was observed as 80% for the rat group with MP-samples and 70% for the rat group with MPC-samples. The histological control of sections from the rat hearts after injection with MPC-samples showed that not only fibers were visible and attached, but some of the cells also turned out to be embedded in the heart tissue (because fluorescence in Figure 5C is provided by LumiTracker Mito dye). Thus, the use of microcarriers improved cell survival in vivo compared to using a conventional cell suspension.

## 3. Discussion

The inability of transplanted myocardial cells to establish a reliable electrical connection with the recipient heart represents one of the main problems in the development of a successful procedure for cardiac muscle regeneration and repair. The manifestation of this problem depends on the method of delivery of cultured cells. The injection of a cell suspension could lead to prolonged ventricular tachycardia during engraftment [8,13], and the attachment of cultured tissue flaps is associated with ventricular tachycardia and delayed excitation waves. This is because gap junctions in the flaps provide less conduction velocity than in the heart [10,16]. Another form of the cell’s synchronization problem is ectopic stimulation of the heart during spontaneous graft activity [13]. The systematization of undesirable side effects from cell engraftment has led to the emergence of a separate term—engraftment arrhythmia. To the best of our knowledge, no single mechanism underlying engraftment arrhythmia has been identified, but the occurrence of such arrhythmias is most noticeable in the first days after cell injection [8,10,13,14]. Despite numerous reports on the significant functional improvement of contractility due to cell engraftment [15], underdeveloped intercellular contacts and potential engraftment arrhythmia reduce the feasibility of cell therapy. The development of new procedures for cardiac tissue regeneration based on the microcarriers proposed in this work should optimize the initial stage of graft–host interaction (in the first hours after contact), improving their synchronization and will be a promising target for future studies with longer follow-up of the positive and negative effects of transplantation.

The synchronization of cell excitation requires that the cells be electrically or mechanically coupled. In this work, we did not completely exclude the possibility of mechanical connection as a way to stimulate cells. Electrical coupling between cardiac cells is thought to be possible through the formation of gap junctions [24,25]. However, an alternative explanation claims that gap junction formation between excitable cells is not obligatory for electrical coupling [26]. Our experiments do not support or discourage any of the points of view above; we are proving the fact that in excitable cells with microcarriers, physical contact with the recipient heart produces calcium releases synchronized with host–tissue excitation, even after a short period (30 min) after transplantation. Given that the organized spread of excitation in the cardiac tissue is a prerequisite for the organized contraction of the heart, this finding might be important for regenerative medicine as evidence that microcarrier-grown and -delivered cardiomyocytes can functionally fuse with the host tissue immediately after contact. Summarizing under the discussion of excitation transfer from the heart to the graft, we can conclude that within this work, we do not explain the underlying mechanism of such rapid cell–tissue synchronization, but we assume that this mechanism must be of a physical (mechanostimulation or EPC) rather than biochemical nature (GJs). This assumption follows from the fact that the synthesis of connexin proteins and their organization into gap junctions in such a time interval seems impossible. High-resolution microscopy (e.g., scanning probe nanotomography [21,27]) and optical mapping of the action potential of both cell grafts and the recipient heart are required to explain the physical transfer of excitation. To the best of our knowledge, this early stage of host–graft interactions (30 min) has not been investigated in vivo for excitation transmission (e.g., with optical mapping). However, existing in vivo studies do not confirm or reject the possibility of synchronization of graft and host cells in such a short time [8,10]. An in vitro study [11] of the electrophysiological host–graft interaction showed the possibility of ephatic synchronization two days after the first contact (not excluding the faster formation of EPC). However, the mentioned study is limited by oversimplification of the spatial organization of the contact spot between the host and the graft—the contact area was limited by the excessive spreading of cells cultured in monolayers, which complicates the formation of an EPC [26]. The formation of an EPC over the same two days was shown in a more realistic in vitro experiment [18] with an increased contact zone due to the transfer of cells on fibroin microcarriers over the monolayer. In the present experiments, we were able to conduct an in vivo simulation of this process and clarify the time frame required for cell synchronization. We have shown that the use of microcarriers and the preliminary restoration of cell excitability can be a sufficient condition for the occurrence of synchronization in the first 30 min.

Regardless of the specific mechanism of excitation transmission, cell coupling requires the presence of contact between cell membranes. In the case of a suspension of cultured cells, the only way to form such a contact spot is through adhesion and spreading, the initiation of which takes on the order of 1 h [9,11]. This fact is used, for example, on the basis of the pre-plating method for purifying cardiomyocyte populations of both NRVMs and iPSC-CMs—pre-plating within 1 h leads to the adhesion of fibroblasts or byproducts of differentiation, respectively, but is insufficient for the adhesion of cardiomyocytes. The same time frame of contact formation between cardiomyocytes was predicted by computer simulations [28]. Repetition of experiments from Section 2.1 with solitary cells without microcarriers apparently resulted in cells not attaching to the heart surface and leaving the seeding site: as in the pre-plating procedure, 30 min is not enough time to even initiate adhesion of a solitary cardiomyocyte. This fact is general and not related to the specific design of the experiment: to confirm this, we examined the attachment of single cells under conditions that are most favorable for cell adhesion. Such conditions are transplantation (seeding) of cells on the surface of a conductive monolayer of cardiomyocytes (immovable and flat, unlike the surface of the heart). Figure S2 shows that seeded cells (highlighted with LumiTracket Mito dye) are not able to restore excitability and synchronize with excitation of the monolayer 30 min after seeding. To be sure, we checked that the formation of coupling occurs, but only 90 min after cell seeding (Figure S2, lower panels and Supplementary Video S4). Thus, the electrical coupling of cells and recipient tissues in 30 min is possible only when using a microcarrier.

The application of microcarriers has opened a new scenario of contact spot formation: fiber coating with the HFN protein leads to the capture of microcarriers by cardiac cells. As a result, the mutual location of the graft and host tissue becomes fixed. At the same time, the cytoskeleton of the graft is already preliminarily flattened and elongated along the fiber. Confocal microscopy of the graft on the surface of the cell monolayer gives us the idea that the cell cytoskeleton retains plasticity and mobility after the moment of contact. Thus, it is possible to apply biodegradable polymers in the composition of the microcarrier, whose degradation will not affect the mechanical stability of the grafts while accelerating their initial attachment. It is worth noting that there is a strategy to use porous microcarriers (instead of fiber ones) suitable for injection [29]. Cardiomyocytes can grow into the pores of such microcarriers. However, the porosity of the polymer [30] in the context of myocardial cell replacement therapy can lead to local tissue anisotropy (for example, elongation of cells along tortuous pores towards the center of the microcarrier or a larger substrate [31]), which is unnatural for the aligned structure of the contractile elements of the heart. In silico studies show that the presence of local anisotropy is a trigger for the formation and maintenance of arrhythmogenic spiral reentry waves [32]. The use of optical tags in the microcarrier composition allowed us to prove the fixation of the graft in the rat heart in vivo for a long time. The estimate of the formation time of ephatic cell coupling obtained in the presented study will be useful for in silico modeling [13] of cardiac cell engraftment. The implementation of in silico studies will speed up and reduce the cost [33] of further development of new therapeutic approaches for cell therapy of cardiac injuries. 

Although the important role of EPC in the synchronous work of the heart is increasingly noted in fundamental studies [13,23,25], it is important to note that the presence of a weak unpredictable transmission mechanism can have a negative effect in the form of increased arrhythmogenicity. The role of stochastic EPC in the formation of EA has been studied in silico [13] and deserves great attention in further studies of cell transplantation on microcarriers. In this paper, we develop the idea [18] that microcarriers give control over the stochastic EPC process, and the formation of an ephatic coupling is at least predictable and may precede the formation of a more reliable coupling mechanism. The study of the EA mechanisms requires the delivery of a larger number of cells capable of overcoming the sink–source mismatch to create an ectopic source of the heart excitation. In this study, the number and density of seeding are regulated by the fact that it is necessary to avoid electrical coupling of NRVMs with each other until the moment of transplantation. Otherwise, the transplanted cell will have two potentially competing sources of excitation—the surface of the heart (this mechanism is being studied in this work) and excitation of the attached cell. The second factor can be mediated by both spontaneous activity and transmission of excitation from the heart. This situation leads to a false-positive interpretation: if there is a chain of several transplanted NRVMs on the heart’s surface (which formed coupling before transplantation), then the presence of at least one sync cell will lead to excitation of the entire chain, but this does not mean that each of them is capable of receive excitation from the heart and be interpreted as a sync cell.

To estimate the critical (maximum) cell density allowing identification of sync and async cells, we increased the cell density by a factor of 1.5, Figure S1A. The activation map shows the transmission of excitation in all cells, respectively, the critical planting density is exceeded. This leads to the conclusion that the cell density used in Section 2.1 (Figure S1B) is close to critical, so increasing the density or number of cells in this work will be contrary to its original goal—to study the coupling of transplanted cells with the recipient heart. However, the proposed method of cell delivery itself does not impose restrictions on the density or number of delivered cells: in Figure S1B, the dotted line shows areas with a high (subcritical) density of excitable cells on the surface of the heart.

There are three negative factors that reduce the effectiveness of graft transplantation: ischemia, due to the absence of vasculature within the injected cell clumps and their delivery into an ischemic environment; anoikis, due to the need to detach these anchorage-dependent cells from their substrate for injection; and inflammation-related factors, such as free radicals, cytokines, and natural killer cells [7]. In the case of iPSC-CMs or other immature cell types, the need for cell maturation after transplantation has also been highlighted [34,35]. The use of microcarriers solves the problem with anoikis. The effect of ischemia in this work was leveled by adhesion to the heart surface (epicardium) washed with perfusate (Tyrode’s solution), but microcarriers do not exclude the possibility of target delivery of substances that reduce the effect. Such substances could theoretically be incorporated into the microcarrier and released as the polymer biodegrades, optimizing cell delivery to the myocardium. Finally, inflammation-related factors are leveled in this work due to the absence of recipient animal blood in the perfusate composition. Summarizing the above, our proposed method of cell delivery can act as an initial configuration for the flexible creation of new methods of cell transplantation for specific cardiac healing tasks with a high rate of cell–tissue synchronization, improved mechanical fixation and noninvasive optical control, and the absence of anoikis of delivered cells.

Limitations: The described study is limited to the transplantation of cells into a healthy rat heart and studying the early stage of cell synchronization. Therefore, based on the results obtained, we could not state whether the new delivery method would have a significant impact on the final result of transplantation (complete engraftment with the formation of all GJ within a few weeks [9]) when compared with existing methods of delivery (especially when delivering a large number of cells comparable to tissue-engineered patches). However, we hypothesize that the availability of rapid cell synchronization will optimize and accelerate the formation of coupling and may reduce the proportion of engraftment arrhythmias [8], which is the main side effect of cardiac cell therapy [1].

Fluo4-AM was used only in transplanted cells to avoid overlapping fluorescence signals from the host and graft and to exclude the false-positive detection of “sync” cells. In principle, these signals are distinguishable with a confocal microscope or higher spatial resolution mapping [18]. In the context of this work, we believe that the spontaneous activity of single grafts is not capable of leading to ectopic excitation of the heart due to sink–source mismatch. The counting of “sync” and “async” clusters was rather a demonstration that both scenarios were observed. The capabilities of the optical setup did not allow mapping and characterizing all the transplanted cells because of the low spatial resolution. It was also hampered by mechanical contractions of the heart, which moved the cells from one focal plane to another, thus also affecting the signal intensity in individual pixels. The calcium traces shown in Figure 3B were obtained in a single focal plane on a section of the heart with minimal displacement during systole, and the fluorescence intensity was measured in a frame (3 pixels × 3 pixels) that moved with the cell during systole. Fluo4-AM dye is not approved by the Food and Drug Administration for clinical or medical use. However, calcium activity can be visualized with viruses that produce fluorescent labels [8] or with FDA-approved water-soluble dyes. In our case, Fluo4-AM was chosen because of its high signal-to-noise ratio, fast staining protocol, and safety in cell culture, even with repeated use [36]. One of the FDA-approved dyes is BODIPY [37]. In this work, we used its analogue BDP with an extended lifetime [38], but its toxicity has been studied to a lesser extent. Finally, the LumiTracker Mito showed a stable signal for two weeks, which indicated the stability of the cell mitochondria in the presence of this dye. In practical applications, this dye can be replaced by the FDA-approved analogues [39] or viruses mentioned above [8].

The experiment with an isolated heart was planned to exclude possible artifacts due to external stimulation of the heart with an electric field. Mostly, the spontaneous activity of the heart, recovered after 30 min in a cardioplegic solution, was recorded. The use of a cardioplegia solution may explain why the heart rate in the recordings with “sync” clusters was insignificantly higher (0.72 Hz against 0.56 Hz, *p* > 0.05). The insufficient washing of the solution can simultaneously explain both the reduced heart rate and the reduced excitability of the grafts.

When analyzing in vivo fluorescence, we were limited to qualitative analysis of fluorescence (i.e., the presence or absence of appreciable fluorescence at the injection site). Quantitative analysis is complicated by two factors (despite the supposed stability of fluorophores). First, we did not fix the posture of the rats during anesthesia. As a result, the same rat appeared in different postures on different images (1 day, 3 days after injection, etc.). Hence, the excitatory light reached the heart in different ways, which affected the absolute value of the signal. Second, we recorded fluorescence with an EMCCD camera in video mode, causing the camera temperature to change during recording and distort the absolute values of the signal. In further work, it would be necessary to improve the methodology of signal registration so that a quantitative assessment of the signal becomes available. In the MPC-sample group, a part of the rats died during surgery, and the percentage of dead rats was 8.5%. We did not perform an autopsy to determine the exact cause of death. As the separate injection of microcarriers and cells did not lead to death in rats, we could only assume that death was not related to the composition of the injection. We did not test the rats for cardiovascular diseases prior to injection, so the case of death during surgery may be related to anesthesia. The rats did not show signs of life after the standard dose of anesthesia, which is possible, for example, in the presence of heart failure before surgery.

Two other limitations are related to in vivo fluorescence. First, we did not account for possible autofluorescence of cardiac molecules (such as two-photon excitation by NADH, NADHp, and possibly FAD), but the control recordings showed no detectable heart fluorescence in the absence of dye. The decreased intensity in the hearts of the MP-sample group may be due to unavoidable photobleaching and dye washout. The rate of photobleaching of the LumiTracker Mito Red CMXR dye used in the C-samples and MPC-samples groups may differ. When imaging cells (C-samples and MPC-samples), dyeing can be replaced by viruses, leading to dye production inside the cell, which will reduce the effect of photobleaching but may lead to unpredictable changes in brightness [17] associated with cell proliferation. 

## 4. Materials and Methods

### 4.1. Polymer Carriers for the Isolation and Transplantation of Single Cardiomyocytes

**Figure 6:**
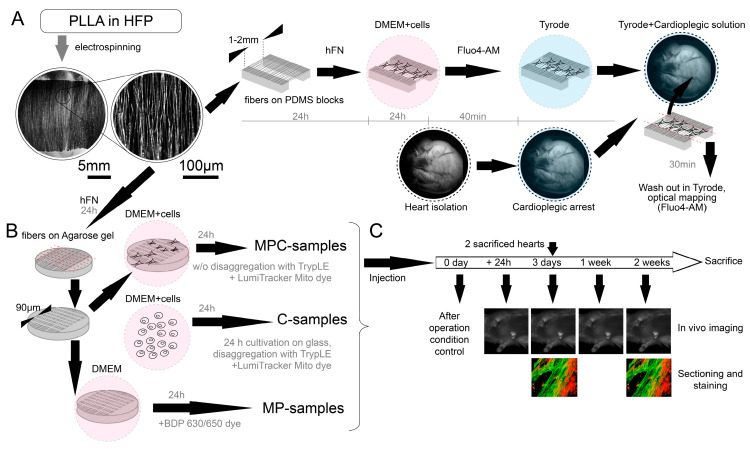
Timeline of experiment workflow. The red dotted lines show where the fibers were cut. Cell culture and cell transfer procedures. (**A**) Timeline of the experiment described in Section 2.1. (Processing of polymer fibers, perfusion of the heart and optical mapping of the grafts with Fluo4-AM dye). Overlay of translucent pink color means placing the sample in the culture medium, blue color—placing the sample in the Tyrode solution. (**B**) Obtaining MPC-samples, C-samples and MP-samples for intramyocardial injections. (**C**) Timeline of in vivo experimental sample testing.

The nanoscaffold fabrication protocol was based on two different methods (Figure 6). The first method (used in Section 2.2) involved the use of agarose, as previously described [18]. Cover slips with a diameter of 15 mm were covered with a 2% agarose (Paneco, Moscow, Russia, Cat# OB0100.050r) solution in water. After solidification, the gel was dried at 70 °C to form a solid agarose film. Aligned electrospun nanofibers on parallel electrodes were located on the film surface. In the electrospinning process, a mixture of 2.5% poly-L-lactid acid PLLA (M_w_ ≈ 700 000, Polysciences Inc., Warrington, PA, USA, Cat# 21,512) and 10% collagen in a 10:1 ratio in hexafluoroisopropanol solvent (HFP, SigmaAldrich Co., Cleveland, OH, USA, Cat# 105,228) was used (Figure 6A). PLLA solution was electrospun using Nanon-01 electrospinning setup (Mecc Co., Fukuoka, Japan). The solution was loaded into the 3 mL syringe and ejected through the 22 gauge blunt tip needle at a flow rate of 0.5–1 mL/h towards the sample collector (aluminum foil with cut 2 cm wide). The voltage applied between the syringe tip and the grounded collector was in the range from 7 to 10 kV and the distance from needle tip to the collector was 10 cm. PDMS (SYLGARD^®^ 184, SigmaAldrich Co., Cleveland, OH, USA, Cat# 761,028) substrate blocks were attached to the drum collector. The electrospinning procedure was carried out for 5 min. The specimens were coated with a solution of HFN (0.16 mg/mL, Gibco, Billings, MT, USA, Cat# 33,016,015) by 24 h incubation at 37 °C to produce a cell adhesive matrix. Next, to prevent fiber detachment during the cell seeding process, we coated them with sucrose water solution (1 g/mL) by spin coating (Instras PDC-2 spin coater (Instras Scientific LLC, Ridgefield Park, NJ, USA), 500–700 rpm, 10 s). Afterward, the immobilized fibers on an agarose film were cut perpendicular to their main direction on a computer-controlled XY table in 90µm increments (Carl Zeiss LSM 710, Carl Zeiss AG, Oberkochen, Germany). The glass with sucrose gel and fragmented fibers was then placed in a Petri dish (35 mm) with agarose coating of the bottom and walls (15 mg per 10 mL of 1× PBS, prepared and dried one day before adding the suspension). Then, 1 mL of cell suspension (concentration of 5 × 10^5^ cells per ml) was added to the dish, and further cell sedimentation took 24 h (Figure 6B). The approximate concentration of kernels per unit area was 500–600 microcarriers per mm^2^. Four samples on agarose gel were used to estimate adhesion efficiency with ImageJ program. In each sample, an area of 0.25 mm^2^ was subjected to analysis: the number of fiber fragments with cells adhered on them was divided into the number of all fiber fragments located in the studied area of 0.25 mm^2^.

For the second method (used in Section 2.1), the algorithm for electrospinning fibers was the same. The spinning of the fibers was on foil rosettes so that the fibers were suspended from the edges of the rosettes. The fibers in this state were then transferred to thin PDMS bars so that the fibers were suspended from these bars. PDMS bars were glued to glass. Then, the fibers were treated with alcohol (70% ethanol) to reduce the charge and the solution with fibronectin for subsequent seeding of cells on them. Only after seeding were the fibers cut using a micromanipulator and a blade for samples with suspended fibers. The difference from the first method is that a section/space with suspended fibers between PDMS blocks (5 mm long, 3 mm wide, the distance between them is about 1–2 mm) is used as a non-adhesive element for cells instead of agarose gel. The resulting structure of PDMS blocks, fibers, and cells adhered to them was peeled off the glass with tweezers and leaned against the surface of the heart—at this moment, the edges of the fibers were cut off from the PDMS blocks, thereby leaving only fibers with cells on the surface of the heart (Figure 6A).

In this study, we used the existing two-day isolation protocol from Worthington-Biochem for neonatal rat cardiomyocyte isolation (http://www.worthingtonbiochem.com/NCIS/default.html (accessed on 5 May 2023)). In brief, hearts were extracted from rat pups (*Rattus norvegicus*, Sprague Dawley breed), aged 1–4 days, and immediately placed in Ca^2+^- and Mg^2+^-free Hank’s Balanced Solution (Gibco, Billings, MT, USA, Cat# 14,180,046) on ice. Only the tissue of the ventricles was isolated—with approximately 50–60% of the initial heart mass being cut off—which included the sinoatrial node, the atria, and the atrioventricular node. The isolated ventricles were minced into small pieces and then left at 4 °C overnight for trypsinization (Trypsin-EDTA 0.25%, Gibco, Billings, MT, USA, Cat# 25,200,056). On the second day, the cells were placed into a collagenase solution (Collagenase type II, 2.25 μg/mL, Gibco, Billings, MT, USA, Cat# 17,101,015) and stirred for 1 h at 37 °C. Next, the suspension of the cells was placed into a T75 flask for 1 h for pre-plating. The cells were then counted with Trypan blue (Gibco, Billings, MT, USA, Cat# 15,250,061), and the concentration of the cells was adjusted to 10^6^ cells/mL.

The isolated cells from the suspension (containing ~70–80% cardiomyocytes) were seeded at a concentration of approximately 2.5 × 10^5^ cells/cm^2^ onto coverslips covered with fibronectin (0.16 mg/mL, Gibco, Billings, MT, USA, Cat# 33,016,015) for further (in next 24 h) disaggregation with TrypLE Express (1×, Gibco, Billings, MT, USA, Cat# 12,605,010) to a unicellular state with making cell suspensions. Some of the isolated cells were seeded on microcarrier samples: with agarose and on suspended fibers. All the samples were cultivated in Dulbecco’s Modified Eagle Medium (DMEM, SigmaAldrich Co., Cleveland, OH, USA, Cat# D6429) with 10% fetal bovine serum (FBS, SigmaAldrich Co., Cleveland, OH, USA, Cat# F4135) for the first 24 h, and then the media was changed to DMEM with 5% FBS. After 3–5 days of cultivation, the samples were examined using an optical mapping approach. Thereafter, basic experiments were conducted. 

For making cell suspensions for injections (C-samples), cardiomyocytes were disaggregated using TrypLE Express (1×, Gibco, Billings, MT, USA, Cat# 12,605,010) to a unicellular state. The resulting cell suspension was marked with a fluorescent tracker LumiTracker Mito Red CMXRos, 575 nm/600 nm (Lumiprobe, Moscow, Russia, Cat# 2251) for approximately 30 min (C-samples and MPC-samples). To make microcarriers, we tracked fibers without cells using boron-dipyrromethene 630/650 amine (Lumiprobe, Moscow, Russia, Cat# 254C0) in MP-samples.

For perfused heart experiments, only the trackers required for optical mapping were used for all transplanted samples (Fluo4-AM dye, Life Technologies, Carlsbad, CA, USA, Cat# F14201) (Figure 6A).

### 4.2. Data Processing and Statistical Analysis

Statistical significance of differences between groups was determined using a one-way ANOVA, followed by Fisher’s least significant difference test for group comparison; the differences were considered significant at *p* < 0.01 or statistically insignificant if *p* > 0.05. 

### 4.3. Perfusion Heart Experimental Protocol

The protocol for isolating the heart and cannulating the aorta began with anesthetizing a lab rat and sacrificing it with a spinal fracture. Next, the heart was carefully removed through the following steps: (1) an incision was made from the xiphoid process to the lateral ends of the edges of the ribs and (2) through the ribs along the left and right anterior axillary lines to provide a cot thoracotomy, and (3) the chest was deviated upward. These steps provided full access to the heart. Sections of the vena cava and aorta completed the procedure for extracting the heart, after which the organ was washed with Tyrode’s salt solution (Sigma-Aldrich Co., Cleveland, OH, USA, Cat# T2145) with heparin and transferred to a Petri dish with the same solution for further manipulations.

The process of attaching the heart to a cannula (a needle with a soft polymer sheath) through the aorta was performed with several turns of surgical thread. From the moment the heart was removed from the body until the start of perfusion, no more than 10 min passed through the cannula.

The perfusion of the heart was carried out using a special installation, according to Langendorf. The setup consisted of two main parts: a perfusion circuit and a recording optical system based on a high-speed imaging setup (Olympus MVX-10 Macro-View fluorescent microscope (Olympus Co., Tokyo, Japan) equipped with a high-speed Andor iXon-3 Camera 897-U (Andor Technology Ltd., Belfast, UK)). The perfusion circuit was prepared for constant circulation (Masterflex L/S Digital Drive, 600 rpm; 115/230 VAC, Masterflex L/S Easy-Load^®^ II Pump Head, SS Rotor; 2-Channel (Cole-Parmer Instrument Company, Vernon Hills, IL, USA)) of a fixed volume of fluid, maintaining the temperature of the perfusate throughout the system (Cole-Parmer Polystat Standard 6.5 L Heated Bath, 150 C, 115 V AC/60 Hz (Cole-Parmer Instrument Company, Vernon Hills, IL, USA)), including the heart chamber, and oxygenating the solution (Cole-Parmer Bubble trap, Water Jacketed Reservoir, Oxygenating Bubbler (Cole-Parmer Instrument Company, Vernon Hills, IL, USA)). The total volume of fluid circulating in the unit was optimized using a compact PDMS polymer heart chamber. The minimum volume that allowed the heart to be perfused with an oxygenated heated solution was reduced to 200 mL. The long-term preservation of the heart was carried out using a special cardioplegic solution, Normacor (CardioSystemPharma JSC, Khimki, Russia). The total cardioplegia time for the hearts was less than 1.5 h. Cardioplegic arrest was performed with Normacor and oxygenated Tyrode’s solution in a 1:4 proportion. For washing the heart after cardioplegia, an oxygenated Tyrode’s solution was used.

### 4.4. Optical Mapping Protocols

For cell culture, optical mapping with Fluo4-AM was performed in Tyrode’s salt solution (pH 7.25 to 7.4) according to the protocol described in [9,40]. The signal was recorded with a 512 × 512 pixels resolution and a sampling frequency of 34 frames per second (Olympus MVX-10 Macro-View fluorescent microscope (Olympus Co., Tokyo, Japan) equipped with high-speed Andor iXon-3 EMCCD Camera (Andor Technology Ltd., Belfast, UK)). The duration and amplitude of the electrode stimulus depended on the tissue culture excitation threshold: from 1 ms to 20 ms duration and from 1 V to 6 V. The stimulation period was 1000 ms, unless otherwise noted (60 ppm). 

For the heart: In general, the mapping protocol for the whole heart was similar to the protocol for cells. We recorded the activity of cells stained with Fluo4 and transferred on fibers to the surface of the heart. We also watched the contractile activity of the heart and recorded it on video (512 × 512 pixels, 34 fps). At the same time, we looked at the spontaneous activity of the heart, as well as the activity of the heart, stimulated by applying an electrode to the apex of the heart (1 Hz). Data processing was fulfilled using the ImageJ program and the associated plugins (http://rsbweb.nih.gov/ij/ (accessed on 5 May 2023)). The calcium traces shown in Figure 3B were obtained in a single focal plane on a section of the heart with minimal displacement during systole, and the fluorescence intensity was measured in a frame (3 × 3 pixels) that moved with the cell during systole (with ImageJ program). The initial location of the frame was chosen manually during diastole, and its location at further time points (in systole) was corrected according to the brightest pixel in the selected frame: the frame was shifted so that the coordinate of the brightest pixel in the 3 × 3 pixel array remained unchanged. To calculate |df/dt|, the difference between adjacent frames in the video was used, normalized to the maximum change between adjacent frames in the video (Figure 3C). ImageJ plugin (time lapse color-coder) was used to build pseudo-3D images and activation maps. Principal component analysis was performed in Python 3, and the original code could be found in the data repository.

### 4.5. Laboratory Animals and Ethical Approval

All procedures were carried out in compliance with the National Institutes of Health Guide for the Care and Use of Laboratory Animals and were approved by the Institutional Animal Care and Use Committee of M. F. Vladimirsky Moscow Regional Clinical Research Institute (protocol No. 4, 4 March 2021) and by the Moscow Institute of Physics and Technology Life Science Center Provisional Animal Care and Research Procedures Committee (Protocol No. A2-2012-09-02).

A total of 44 rats participated in the experiments: 4 for perfusion and 40 for operations. The success rate of all operations was more than 97%; only one rat died during the operation. All rats were anesthetized using the RWD R500 Small Animal Anesthesia Machine using oxygen and isoflurane. No rats were anesthetized more than once a day. In total, there were three groups of rats that underwent surgery, in accordance with the injections administered to them: 10 rats for C-samples, 15 rats for MP-samples, and 15 rats for MPC-samples. 

The experimental protocol for checking animals after the introduction of the suspension occurred within two weeks from the moment of operation. A day after the injection, in vivo imaging was performed. On the third day after the operation, the first animal sacrifice was made, and samples were taken for sectioning and microscopy analysis. In vivo mapping was also carried out on the third day, one week and two weeks after the injection. All rat hearts were examined by fluorescence microscopy and sectioning after two weeks.

### 4.6. Intramyocardial Injection

Access to the heart was carried out through a left thoracotomy. The solution was injected with an insulin syringe containing 2 mL of the sample. Three types of samples were injected: cell suspension (C-samples, about 50,000 cells in 1 mL), microcarriers with protein (MP-samples, contains about 50,000 microcarriers in 1 mL), and microcarriers with cells seeded on them (MPC-samples, about 50,000 microcarriers and estimated 25,000 adhered cells for ~50% seeding efficiency in 1 mL). After injection of the solution into the myocardium, the wound was sutured in layers. Air aspiration from the pleural cavity was mandatory (Figure 6B,C).

### 4.7. In Vivo Imaging

In vivo imaging was performed with a LumoTrace FLUO bioimaging system [41,42] (LumoTrace^®^ FluoEM, Abisense LLC, Sochi, Russia), as follows: rats were anesthetized according to the experiment timeline (Figure 6C) after injection of one of the sample’s types and imaged with fluorescence excitation for fiber-marked samples at λ_ex_ = 630 nm and a 655 nm/40 nm filter or λ_ex_ = 575 nm and for cell-marked samples in a 600 nm/40 nm filter.

### 4.8. Immunofluorescent Staining and Sectioning

After the experiments, all hearts were fixed with paraformaldehyde and sectioned. Cross-sections of the hearts with a width of 30–60 μm were prepared on a cryotome (Thermo Shandon Cryotome FE, Thermo Fisher Scientific, Waltham, MA, USA).

Immunofluorescent staining was performed as follows. Samples were fixed for 10 min in 4% paraformaldehyde (Sigma-Aldrich, Cleveland, OH, USA, 158127), permeabilized for 10 min in 0.4% Triton-X100. Cells were further incubated for 30 min in blocking buffer (1% bovine serum albumin in phosphate-buffered saline, PBS), overnight at 4 °C with primary antibodies and for 1 h at room temperature with secondary antibodies. Cells were washed twice for 15 min in PBS. Nuclei were stained with DAPI (Vector Laboratories, Inc., Brockville, ON, Canada). Samples were analyzed and processed on a Zeiss LSM 710 confocal microscope with Zen black 3.0 software (Carl Zeiss AG, Oberkochen, Germany). Primary antibodies (working dilutions—1:100): sarcomeric α-actinin mouse (Abcam, Cambridge, UK, Cat# ab9465). Secondary antibodies (Thermo Fisher Scientific, Waltham, MA, USA, working dilution—1:400)—Alexa Fluor 568 goat anti-mouse IgG (HþL) highly cross adsorbed (A11031). Three-dimensional imaging of cell monolayers (Figure 4B) was performed for 3 different samples.

## 5. Conclusions

Our experiments showed that molecular machines based on PLLA nanofibers act as sufficient scaffolds for solitary cardiomyocytes during cell transfer in vivo. The key consequence was the restoration of cell excitability by the time of contact with the recipient tissue in the absence of a full-fledged polymeric substrate, thereby combining previously incompatible aspects. This use of polymeric biomaterials led to a unique result: on the isolated rat heart, we demonstrated the synchronization of transplanted cells with the host’s contractions 30 min after the first contact, which also led to the synchronization of transplants with each other. The generally accepted estimate was that the process of electromechanical host–graft synchronization would require several hours or days. Thus, a non-invasive targeted delivery of cardiac cells using nanofiber carriers is proposed.

## Figures and Tables

**Table 1:** Summary table of experimental repetitions.

Experiment	Heart Rate after Wash Out	Sync (Total: 22/46)	Async (24/46)
Heart #1 (Wistar, male, 248 g)	0 Hz (no systole)	0 (no systole)	9
Heart #2 (Wistar, male, 310 g)	0.78–1.28 Hz	5	2
Heart #3 (Wistar, male, 225 g)	0.34–0.51 Hz	11	7
Heart #4 (Wistar, male, 274 g)	0.51–0.68 Hz	6	6

## Data Availability

Raw data (optical mapping and confocal microscopy) and processed data could be found at Data Repository https://doi.org/10.5281/zenodo.7890101, https://zenodo.org/record/7890101#.ZFU4knZBxPY (accessed on 5 May 2023).

## Supplementary Materials

The supporting information can be downloaded at: https://www.mdpi.com/article/10.3390/ijms241210406/s1.
