# Supplementary material for: Novel Molecular Vehicle-Based Approach for Cardiac Cell Transplantation Leads to Rapid Electromechanical Graft–Host Coupling
Source: Int J Mol Sci. 2023 Jun 20;24(12):10406. doi: 10.3390/ijms241210406 (PMC10299342; doi:10.3390/ijms241210406)
Supplement: Supplementary file 1 [file ijms-24-10406-s001.zip › ijms-2410118-supplementary.pdf]

International Journal of
Molecular Sciences
MDPI

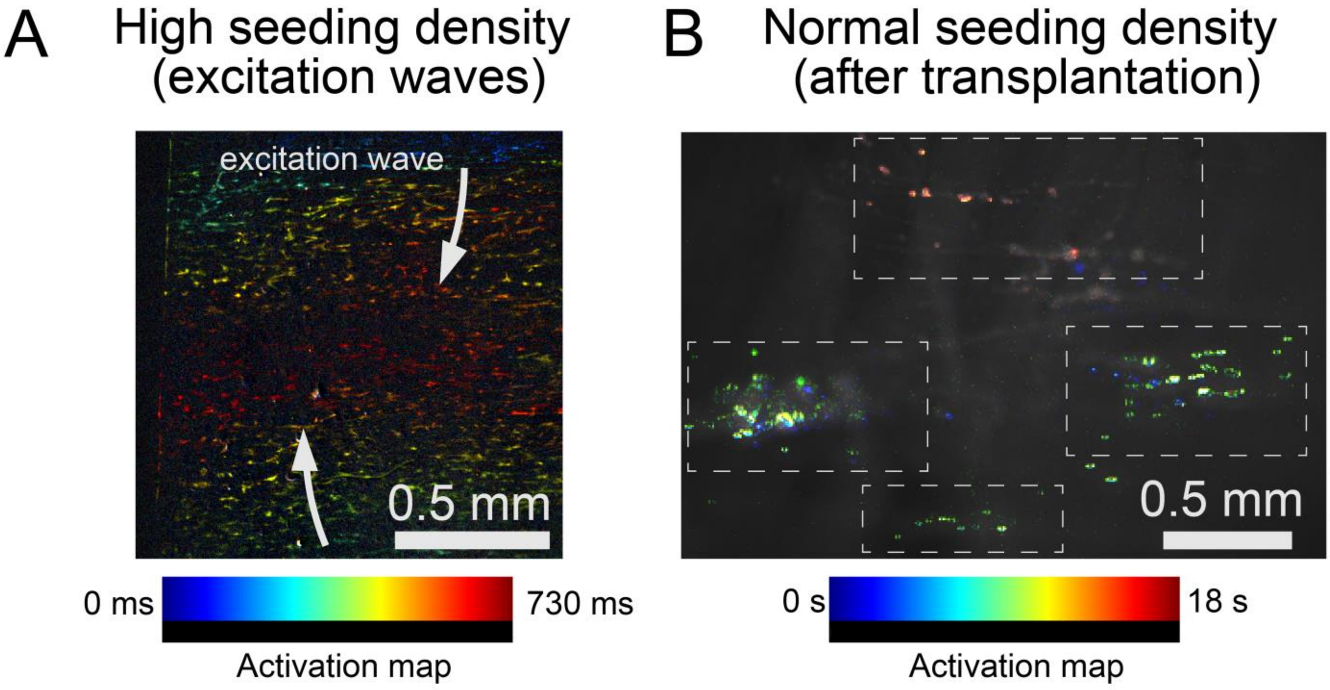

Figure S1. A. Activation map of the conduction of the excitation wave through cells on nanofibers. Obtaining this sample is similar to the sample from Figure 2A, differing only in the density of seeding cells, increased by 1.5 times. All cells of the sample are involved in the conduction of excitation waves. B. Activation map of spontaneous activity of transplanted cells on the surface of the recipient heart (Heart #3). The white dotted line indicates subcritical cell density (i.e., no excitation wave transmission occurs between cells).

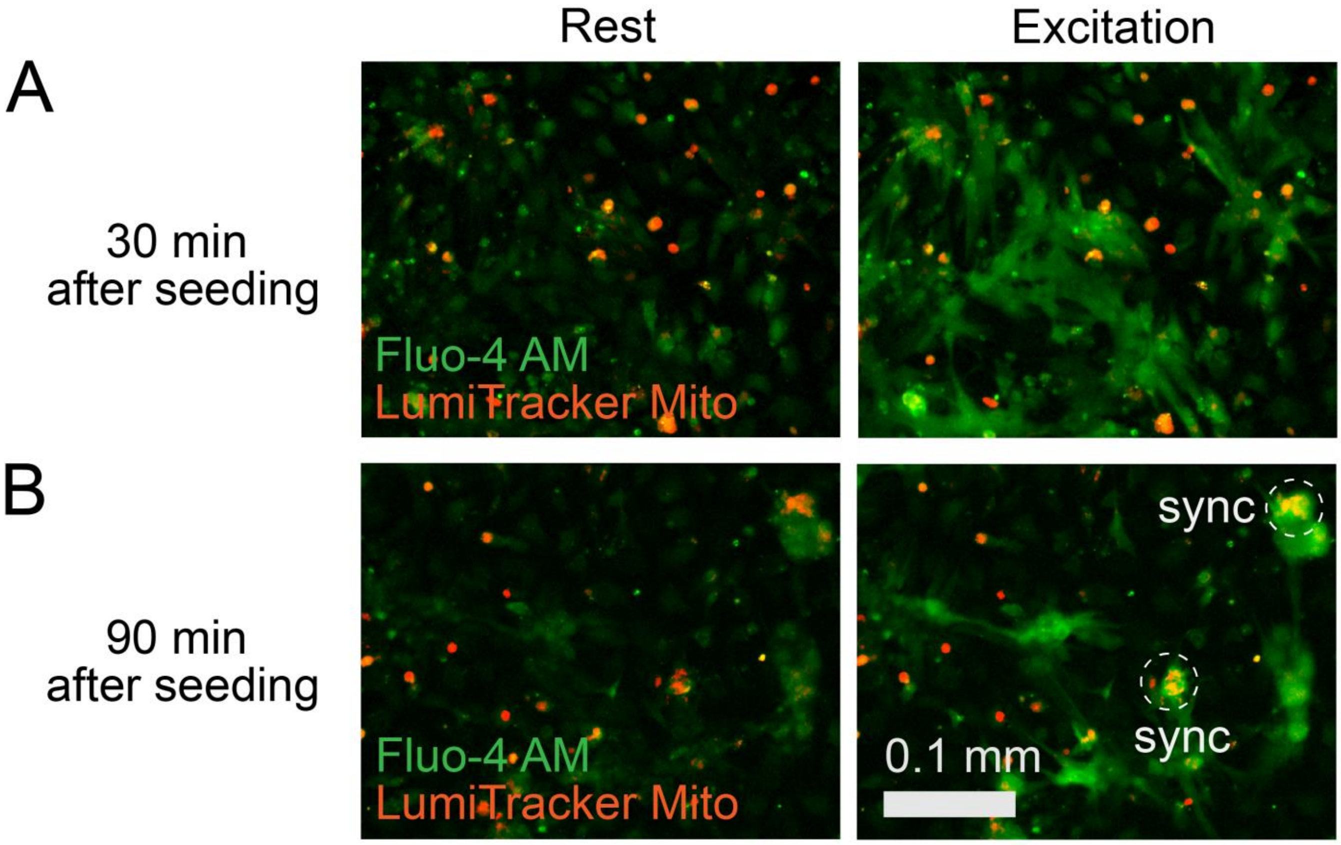

Figure S2. A. Optical mapping of cells with LumiTracker Mito seeded on a conductive monolayer (planting scheme similar to that in Fig. 4B but in the absence of a microcarrier in the cells) 30 minutes after seeding. The image illustrates one of 3 repetitions of the experiment, in none of them synchronization of grafts and monolayer was found. B. Optical mapping at 90 minutes, detection of the first synchronized cells (3 repetitions were used and presented in Data Repository).

Video S1. Conduction of the excitation wave through NRVMs on nanofibers (visualized with Fluo-4 AM). Obtaining this sample is similar to the sample from Figure 2A, differing only in the density of seeding cells, increased by 1.5 times;

Video S2. Optical mapping of Async transplant - in this example, the excitation of the cell occurs only once per recording and does not coincide in time with any of the three systoles of the heart (video is played at 0.5x speed);

Video S3. Optical mapping of Sync transplant - in this example, the excitation of the cell is synchronized with all three systoles presented in the recording. Separately, spontaneous cell contractions between systoles are signed (video is played at 0.5x speed);

Video S4. Optical mapping of NRVMs with LumiTracker Mito seeded on a conductive monolayer (planting scheme similar to that in Fig. 4B but in the absence of a microcarrier in the cells) 90 minutes after seeding, Sync cells are highlighted with white circles.
